# The protocol for a multicentre prospective randomized noninferiority trial of surgical reduction versus non-surgical casting for displaced distal radius fractures in children: Children’s Radius Acute Fracture Fixation Trial (CRAFFT) protocol

**DOI:** 10.1302/2633-1462.65.BJO-2024-0260

**Published:** 2025-05-23

**Authors:** Daniel C. Perry, Juul Achten, James Mason, Daphne Kounail, Nicolas Nicolaou, David Metcalfe, Mark Lyttle, Elizabeth Tutton, Duncan Appelbe, Phoebe Gibson, Matthew L. Costa

**Affiliations:** 1 University of Liverpool, Liverpool, UK; 2 Oxford Trauma and Emergency Care (OxTEC), Nuffield Department of Orthopaedics, Rheumatology and Musculoskeletal Sciences (NDORMS), University of Oxford, Oxford, UK; 3 Warwick Clinical Trials Unit, University of Warwick, Coventry, UK; 4 Oxford Clinical Trials Research Unit (OCTRU), Centre for Statistics in Medicine (CSM), Nuffield Department of Orthopaedics, Rheumatology and Musculoskeletal Sciences (NDORMS), Oxford, UK; 5 Sheffield Children’s Hospital, Sheffield, UK; 6 Emergency Research Oxford (EMROx), John Radcliffe Hospital, Oxford, UK; 7 Bristol Children’s Hospital, Bristol, UK; 8 Independent Lay Member, Liverpool, UK

**Keywords:** Distal radius fracture, Fracture fixations, Surgical casts, Patient-reported Outcomes Measurement Information System, Upper limb, Fractures of the distal radius, surgical casting, Patient-Reported Outcomes Measurement Information System, Upper Extremity, Fracture Fixation, Orthopaedic Surgery, clinicians, plaster cast, EuroQol EQ, wrist fractures

## Abstract

**Aims:**

The remarkable capacity for distal radius fractures in children to remodel raises questions about the necessity and extent of the intervention required to achieve anatomical alignment. The British Society of Children’s Orthopaedic Surgery prioritized this uncertainty as one of their most important research questions. This is the protocol for a randomized, controlled, multicentre, prospective, noninferiority trial of non-surgical casting versus surgical reduction for severely displaced fractures of the distal radius in children: the Children’s Radius Acute Fracture Fixation Trial.

**Methods:**

Children aged four to ten years old inclusive, who have sustained a severely displaced distal radius fracture, are eligible to take part. Baseline function using the Patient-Reported Outcomes Measurement Information System (PROMIS) Upper Extremity Score, pain measured using the Wong-Baker FACES Pain Scale, and quality of life (QoL) assessed with the EuroQol five-dimension questionnaire for younger participants will be collected. Each patient will be randomly allocated (1:1), stratified using a minimization algorithm by centre, fracture type at presentation (completely off-ended or incompletely off-ended), fracture location (metaphyseal or physeal), and age group (four to six years or seven to ten years) to either a regimen of non-surgical casting or surgical reduction.

**Conclusion:**

At six weeks, and three, six, and 12 months, data on function, pain, QoL, cosmesis, and satisfaction with care will be collected. After completion of the main phase of the study, patients will be followed up for a further two years. Up to one year after randomization, the main outcomes plus data on complications, resource use, and school absence will be collected. The primary outcome is the PROMIS Upper Extremity Score at three months post-randomization. All data will be obtained through electronic questionnaires completed by the participants and/or parents/guardians.

Cite this article: *Bone Jt Open* 2025;6(5):560–568.

## Introduction

Fractures of the distal radius and ulna are the most common fractures in children,^1^ and constitute around half of childhood long-bone fractures seen in emergency departments. Most are relatively minor, with some not requiring any treatment,^2^ and others requiring a simple plaster cast. However, intervention is the mainstay of treatment for more displaced distal radius fractures to restore alignment of the bones.

Realignment of the bones is typically performed in the operating theatre under general anaesthesia, although it is sometimes performed with sedation in other clinical settings. During the procedure, external manipulation is performed to realign the bones, which may be followed by the insertion of metal pins or plates, after which the bones are held securely in a plaster cast. This treatment carries the risks and costs associated with admission and anaesthesia, and complications related to the surgery. Metal pins are most commonly used to hold the bone, but these result in complications in around one-third of cases.^3,4^ Complications include infection, injuries to surrounding nerves, and further surgery (approximately 7%).^5^ The use of pins and plates also necessitates later procedures to remove these implants.

While operative treatment is the mainstay of treatment for displaced fractures, it is widely recognized that this may not be necessary. Children’s bones are different to adult bones, principally because the bones are still growing. Growth of the bone arises from specific regions at the ends of a bone, called the physis or ‘growth plate’. As well as enabling growth, the physis also enables a process called ‘remodelling’.^6^ Remodelling allows deformity at or near the physis to correct with growth, with the possible correction of angulation, length, and translation.

As early as 1935, it was shown that fractures near the physis have a huge potential for remodelling in children.^7^ The rate of remodelling at the wrist is exponential, with the most marked remodelling in the early months following a fracture and little or no improvement seen beyond three years. Remodelling occurs most markedly in children with the most remaining growth, with studies consistently demonstrating good results among children aged up to 11 years.^8-10^ Studies have shown that, amongst children aged up to 11 years old, even the most severe ‘completely off-ended’ metaphyseal fractures remodel with no residual functional impairment.^11,12^

There is considerable controversy about how best to manage these fractures, with surgeons recognizing the potential for remodelling, but expressing unease about whether the bone will correct itself, and uncertainty about how families will react to waiting for the initial bent appearance of the child’s arm to improve.^11^ This was made one of the top research priorities by the British Society of Children’s Orthopaedic Surgery.^13^ Clinicians in the UK, and internationally, now feel compelled to strengthen this evidence.^14^ This is the protocol for a multicentre prospective randomized noninferiority trial of surgical reduction versus non-surgical casting among children with displaced fractures of the distal radius.

This is an abridged version of the protocol for broad transparency; the full working protocol and iterative developments are available on the National Institute for Health and Care Research (NIHR) website.^15^

## Aims

The aim of this pragmatic randomized controlled trial is to evaluate the clinical and cost-effectiveness of non-surgical casting, compared with surgical reduction for the treatment of severely displaced fractures of the distal radius in children.

The primary objective is to determine whether non-surgical casting is noninferior to surgical reduction, measured using observed differences in the Patient-Reported Outcomes Measurement Information System (PROMIS) Upper Extremity Score^16^ at three months post-randomization.

The secondary objectives are:

– to quantify and draw inferences from differences in function using the PROMIS Upper Extremity Score between trial treatment groups during the first year after randomization;
– to quantify and draw inferences from observed differences in pain using the Wong-Baker FACES Pain Scale^17^ between trial treatment groups during the first year after randomization;
– to quantify and draw inferences from observed differences in quality of life using the youth version of the EuroQol five-dimension questionnaire (EQ-5D-Y)^18^ between the trial treatment groups during the first year after randomization;
– to determine the complication rate up to one year after randomization, including refracture, the need for further operative fixation, and the absence of radiological remodelling;
– to estimate the cost-effectiveness of the treatments to the NHS and the broader economy, up to one year after randomization;
– to quantify and draw inferences from parental satisfaction with the cosmetic appearance of the arm between trial treatment groups during the first year after randomization;
– to quantify and draw inferences from parental satisfaction with care between trial treatment groups during the first year after randomization;
– to quantify and draw inferences from school attendance between trial treatment groups during the first year after randomization;
– to determine the impact of injury, treatment, and recovery on parent and child experience of daily life and the outcomes that are important to them; and
– to determine the barriers and facilitators to trial recruitment from parent/child and staff perspectives.

Long-term outcomes, to be reported separately, are: to quantify and draw inferences from longer-term pain; function; and complications annually up until three years after randomization.

A schedule of data collection is outlined in Table I.

**Table I.** Outcomes collection schedule.

Timepoint	Data collection
Pretreatment (clinic)	PROMIS UE baseline, Wong-Baker FACES Pain Scale, EQ-5D-Y
Removal of immobilization (clinic)	Complications
Six weeks	PROMIS UE, Wong-Baker FACES Pain Scale, EQ-5D-Y, VAS cosmesis, complications, school attendance, and economics questionnaire
Three months	PROMIS UE, Wong-Baker FACES Pain Scale, EQ-5D-Y, VAS cosmesis, complications, school attendance, and economics questionnaire
Six months	PROMIS UE, Wong-Baker FACES Pain Scale, EQ-5D-Y, VAS cosmesis, complications, school attendance, and economics questionnaire
One year	PROMIS UE, Wong-Baker FACES Pain Scale, VAS cosmesis, satisfaction score, EQ-5D-Y, complications, school attendance, and economics questionnaire
One year (recruitment centre)	All routinely available radiographs documenting the course of this injury will be collected from the patient record
Two years	PROMIS UE, Wong-Baker FACES Pain Scale, EQ-5D-Y, VAS cosmesis, complications
Three years	PROMIS UE, Wong-Baker FACES Pain Scale, EQ-5D-Y, VAS cosmesis, complications. Any further routinely available radiographs documenting the course of this injury will be collected from the patient record

EQ-5D-Y, youth version of the EuroQol five-dimension questionnaire; PROMIS UE, Patient-Reported Outcomes Measurement Information System, Upper Extremity; VAS, visual analogue scale.

We explored potential outcomes with the parents and children who advise our research. Children believed that early return to normal function was most important to them, and therefore the function at three months was chosen to be the primary outcome. Given the participants’ age, all outcomes will be proxy-reported by the parents, except for pain, which will be child-reported.

## Summary of outcomes

### PROMIS Upper Extremity Score for Children

PROMIS is a well-validated assessment of upper limb function in children, encompassing activities of daily living, participation in school activities, and hobbies. This outcome is used in other studies funded by the NIHR Health Technology Assessment (HTA) programme, including SCIENCE^19^ and FORCE.^20^ In general, ‘PROMIS scores’ are a collection of patient-reported health status tools available for children and adults that were developed in collaboration with the USA National Institutes for Health. The PROMIS Paediatric item banks were developed using a strategic item-generation methodology adopted by the PROMIS Network utilizing item response theory. Field-testing occurred among 4,129 children aged eight to 17 years. Lower T-scores indicate a worse outcome for upper limb function. PROMIS is available in full (30 questions), as a short-form (eight questions), or as a computer-adaptive test (CAT) with around eight questions. A CAT enables the answer from one question to inform the choice of the next, and so each participant could answer a distinct set of questions to arrive at their score. The CAT version will be used in this trial.

The PROMIS Upper Extremity Score for Children has been demonstrated to have convergent validity with other tests used in the assessment of upper limb function in children with congenital limb abnormalities,^21^ Disabilities of the Arm, Shoulder and Hand Score (DASH)^22^ (r = 0.80; p < 0.001), and Paediatric Outcomes Data Collection Instrument (PODCI)^23^ (r = 0.70; p < 0.001). DASH is an adult measure of upper limb function with items that lack face validity among children (DASH S/PA Module is distinct from the general DASH outcome tool), and PODCI is a general measure of disability. The PROMIS Upper Extremity Score for Children correlates better with physiological tests of upper limb function (grip strength and pinch strength; r > 0.5; p < 0.050),^21^ than these other patient-reported measures.

Although PROMIS enables self-reporting among participants from eight years of age, this trial will use parent-reported function among all age groups, given the varying age of participants.

### Wong-Baker FACES Pain Scale

The Wong-Baker FACES Pain Scale is a validated self-reported tool that will be self-reported among all children in the study. It is an ordinal assessment of pain outcomes, using a series of six facial expressions to illustrate the degree of pain intensity. A numerical rating is assigned to each face (from zero, ‘no hurt’, to ten, ‘hurts worst’). It has been validated for use among children over three years old, including in the Emergency Department setting. It is particularly useful among younger children, as only one-third of children aged five to 14 years understand the concept of a visual analogue scale (VAS).

### EQ-5D-Y

The EQ-5D-Y is the youth version of the EuroQol five-dimension three-level questionnaire (EQ-5D-3L), which is a validated, generalized, health-related quality of life questionnaire consisting of five domains related to daily activities, each with a three-level response. EQ-5D-Y has been especially adapted in terms of language for use among children, with both proxy and self-reported versions.^18,24^ Given the age of participants within this trial, as with the PROMIS tool, we plan to use the proxy-reported version throughout. There is currently ongoing work, to produce EQ-5D-Y value sets for use in children and adolescents. Our interim solution is to apply adult EQ-5D value sets to the EQ-5D-Y classification, but to use the EQ-5D-Y valuation system if this is ready before the Children’s Radius Acute Fracture Fixation Trial (CRAFFT) trial is complete. Utility valuations in the York A1 tariff set range from no problems in any of the five dimensions in the EQ-5D descriptive system (value = 1.0) to severe or extreme impairment in all five dimensions (value = −0.594).^25^

### Complications

All complications will be recorded, but particular note will be made of complications related to the cast (including but not limited to pressure areas) or surgery (including but not limited to wound infection, nerve injury, and scar problems (over-granulation/hypertrophy/keloid)), and the need for further unplanned surgery in either group (including surgery for revision, refracture, or broken metalwork). Planned surgery for the removal of metal pins/screws/plates will be recorded as part of routine treatment, and will not be regarded as a complication. Any digital images of the wrist that have been collected as part of routine practice will be collected from the Picture Archiving and Communication System (PACS) at one year after randomization. A further collection of routinely taken images during long-term follow-up will be collected at three years after randomization. No specific imaging is required at any stage for research purposes. Where available, the images will be used to assess the degree of residual deformity. The collection of routine digital images is not any different to standard care for the purposes of the Radiation Assurance Consistency Review Guidance run by the Health Research Authority.^26^

### Cost-effectiveness

Resource use and quality-of-life data will be collected prospectively over the first year of patient follow-up. A UK NHS and Personal Social Services (PSS) perspective will inform the primary analysis. A broader social perspective will include out-of-pocket expenses, parental absence from work, and any periods of school absence.

### Parent assessment of cosmesis and satisfaction with care

The perception of cosmesis will be collected using a VAS, and satisfaction with care using a Likert scale.

### School attendance

The number of days absent from school due to the injury and the resulting treatment.

## Methods

Children will be eligible for inclusion into the trial if:

- there is radiological evidence of a severely displaced wrist fracture, at or adjacent to the physis (Salter-Harris type II^27^ or a metaphyseal fracture); with or without a corresponding ulna fracture;
- the treating clinician believes that they may benefit from surgical reduction with or without fixation; and
- they are aged between four and ten years, inclusive.

Children will be excluded from this trial if:

- the injury is more than seven days old;
- the injury is part of a more complex wrist fracture (i.e. open or fracture extending into the joint);
- there are other fractured bones elsewhere in the body, in addition to the affected wrist injury; or
- there is evidence that the patient and/or parent would be unable to adhere to trial procedures or complete follow-up, such as insufficient English language comprehension, developmental delay or a developmental abnormality, or no access by parents to mobile data/internet.

### Consent

Recruitment will take place in more than 32 NHS trusts that treat children with this injury in the UK. Eligible patients will be identified by the clinical team. A member of the local research team will present the patient and parents/guardian with age-appropriate participant information sheets or online study information and verbal explanation of the trial procedures. The patient/parent/guardian will then be given the opportunity to discuss any issues related to the trial with a member of the local research team and members of their family and friends.

The parent/guardian will then be asked to sign an electronic informed consent form and, where appropriate as assessed by a member of the local research team in collaboration with the parents, children will be asked for their assent. The absence of assent does not exclude the patient from the study if consent has been obtained from the parent/legal representative. However, if a child completes the assent form indicating that they do not wish to participate, the child will not be included in the study. A copy of all electronic consent and assent forms will be emailed to the parent directly. If the parent does not have an email address, the local research team will download a paper copy of the completed consent/assent forms to give to the parent.

### Randomization

The patient will be randomized after consent and baseline data has been obtained, either in the Emergency Department, or at the first assessment in the fracture clinic. All hospital treatment areas have access to the internet so will access the randomization service in real time, i.e. there will be no delay to patient treatment. Consented participants will be randomized to one of two intervention groups (1:1) using a computer randomization service provided by the Oxford Clinical Trials Research Unit (OCTRU). Randomization allocation will be implemented using a minimization algorithm with stratification factors: centre, type of fracture translation (completely off-ended versus incompletely off-ended), fracture location (metaphyseal or physeal), and age group (four to six years, seven to ten years). The minimization algorithm will be seeded with a number of allocations, and a non-deterministic probabilistic element will be introduced to prevent predictability of the treatment allocation.

Stratification by centre will help to ensure that any clustering effect related to the centre will be equally distributed between the trial groups. Each hospital has a children’s injury unit dealing with these wrist fractures on a daily basis. All of the recruiting hospitals, and all orthopaedic units throughout the NHS, use these techniques as part of their normal fracture management practice, so staff will already be equally familiar with both forms of treatment. This cannot eliminate the clinician-specific effect of an individual at any one centre. However, as the procedures are commonplace across the NHS, many clinicians (15 to 30 at each centre, including consultants and trainees) will be involved in the management of this group of patients. We therefore anticipate that each individual clinician will only treat a handful of those enrolled in the trial, which greatly reduces the risk of a clinician-specific effect on the outcome in any one centre.

### Blinding

Patients and their parents/guardians cannot be blinded to their treatment. The treating clinician will of course, not be blinded to the treatment they are providing. The outcome data will be collected directly from the patient and/or their parents/guardians. Outcome assessors will be blinded to the participant’s treatment allocation.

## Trial treatments

This trial will compare two approaches to treat displaced distal radius fractures in children aged four to ten years, inclusive.

### Non-surgical casting

This technique involves the application of a plaster cast to hold the bone fragments in the most optimal possible position, without giving medication to deliberately alter the conscious level of the child. This may be the initial plaster cast used to stabilize the fracture, or the plaster cast may be changed by the clinician to maximize patient comfort and fracture stability. Although the principles of applying a plaster cast are inherent in the technique, in this pragmatic trial, the type of casting material, extent of the cast, and the details of the technique will be left to the discretion of the treating clinician as per their usual technique. A record will be made of the cast details and any cast changes. Usual practice is for the plaster cast to be used for four to six weeks.

### Surgical reduction

Surgical reduction with or without fixation will be performed. The bones will be realigned under general anaesthesia or sedation altering the conscious state of the child. The method used to hold the bones in position will be at the discretion of the clinician (i.e. plaster cast alone, plaster cast and wires, or plaster cast and plate). A record will be made of the operative details, the cast details, and any cast changes. Following surgery, the usual practice is for the arm to be immobilized in a cast for four to six weeks. Specific details on the techniques and materials used in theatre will be collected for each participant.

### Rehabilitation

In this pragmatic trial, rehabilitation will be left to the discretion of the treating clinicians. However, a record of any rehabilitation input (type of input and number of additional appointments), together with a record of any other investigations/interventions, will be requested as part of the six-week, three-, six-, and 12-month follow-up.

### Adverse event management

Serious adverse events (SAEs) will be entered onto the SAE reporting form and reported to the central study team. Once notified, causality and expectedness will be confirmed by the chief investigator or trial-nominated clinician. Some adverse events that are foreseeable as part of the proposed treatment will not be reported on a SAE reporting form; they will instead be recorded on a complications reporting form. These events include: 1) complications related to the cast (including but not limited to pressure areas); and 2) complications related to surgery (including but not limited to wound infection, nerve injury, scar problems (over-granulation/hypertrophy/keloid)), pain, and the need for hospital admission to manage these complications, or further unplanned surgery in either group (including surgery for revision, refracture, or broken metalwork). Planned surgery for the removal of metal pins/screws/plates will be recorded as part of routine treatment, and will not be regarded as a complication.

The end of the trial will be defined as the collection or receipt of the last follow-up questionnaire from the last participant.

### Sample size

A total of 674 participants providing data on the PROMIS Upper Extremity Score for Children at three months after randomization (337 in each group) will provide 90% power and 2.5% (one-sided) significance to detect whether non-surgical casting for the treatment of displaced wrist fractures is noninferior to surgical reduction, assuming a noninferiority margin of −2.5 points, a SD of 10, and no difference between groups (PASS 16 Power Analysis and Sample Size Software (2018); NCSS, USA). The choice of the noninferiority margin and the baseline SD was based on discussions with patients, their parents, and the literature validating the PROMIS Upper Extremity Score in a range of different diseases. Allowing for 10% loss to follow-up, this yields an overall target of 750 patients (375 per group).

Raw scores of the PROMIS Upper Extremity Score for Children are translated into standardized T-scores with a population mean of 50 and a SD of 10. The minimal clinically important difference (MCID) for the PROMIS Upper Extremity Score among children with mild forms of disability has been demonstrated to be three or four.^28^ In general, the bank of paediatric PROMIS measures have an MCID of three points, in a range of different diseases.^29^ We worked with parents with upper limb injuries to rescore the PROMIS Upper Extremity tools for the vignettes to indicate ‘the minimum difference that is likely to be noticeable’, and ‘the minimum difference that would be necessary to justify undertaking surgery’. Although a score of four points appeared to be the minimal difference noticeable to parents, the clinically important difference required to justify surgery was five points (standardized effect size > 0.5). Parents and children demanded a larger effect size to justify the intervention of surgery. Other studies have similarly highlighted that patients often seek greater effect sizes than the established MCID to warrant surgical interventions.^30^ A noninferiority margin of 2.5 points was decided upon as half the maximum tolerated reduction in acceptability to patients and their parents to justify surgery. If non-surgical casting is shown to be noninferior based on this, then the results are likely to change clinical practice for these fractures.

For noninferiority, the lower 95% CI of the treatment difference between non-surgical casting and surgical reduction is assessed against the noninferiority margin of −2.5 points and, if it lies above this, then non-surgical casting will be found to be noninferior to surgical reduction. If noninferiority is shown, then superiority will also be tested at the 2.5% (one-sided) significance level. In this case, the lower 95% CI would be above zero points.

As the degree of translation has the potential to influence outcome, we have incorporated this as a stratification factor to ensure that it is balanced across the treatment groups, and we will assess for differential outcomes in the important sub-groups using treatment-by-sub-group interactions. From the site audits, approximately one-quarter to one-third of these fractures will be completely off-ended. This implies that from the 750 total patients recruited, 200 to 250 patients will have completely off-ended displaced fractures. This is an important sub-group for surgeons as this represents the most severe fractures. Collecting 200 patients within this sub-group will enable noninferiority for non-surgical casting with surgical reduction assuming 90% power, 2.5 (one-sided) significance with a noninferiority margin of between −4.5 and −5 points on the PROMIS Upper Extremity Score for Children at three months, assuming a SD of 10. This is above the maximum tolerated reduction in acceptability to patients and their parents to justify surgery. We therefore plan on continuing recruitment until a minimum of 200 patients in the completely off-ended sub-group have been randomized. Fracture location (metaphyseal compared with physeal) and participant age also has the potential to influence outcome, and we have also incorporated these as stratification factors to ensure that these are balanced across the treatment groups.

### Analysis population

The primary analysis of the primary outcome will be based on the intention-to-treat (ITT) population. This will include all randomized participants with available data who will be analyzed according to their allocated intervention, regardless of the treatment received.

US Food and Drug Administration regulations recommend that an analysis is performed both as randomized (ITT) and as treatment received (per protocol; PP), aiming to demonstrate noninferiority. Therefore, a secondary analysis will be undertaken on the PP population, which will include all patients who received their allocated treatment and did not have any major protocol deviations. Major protocol deviations will be finalized following a blinded review of the data prior to the primary outcome analysis datalock.

All analyses of the secondary outcomes will be performed for the ITT population.

## Analysis

A separate statistical analysis plan (SAP) with full details of all statistical analyses planned for the data of this study will be made public prior to the study datalock. The SAP was reviewed and received input from the Trial Steering Committee (TSC) and Data Safety and Monitoring Committee (DSMC). Any changes or deviations from the SAP will be described and justified in the protocol, final report, and/or publications, as appropriate. It is anticipated that all statistical analyses will be undertaken using Stata (StataCorp, USA) or other well-validated statistical packages.

Standard descriptive statistics will be used to describe the demographics between the treatment groups reporting means and SD or medians and IQR as appropriate for continuous variables and numbers and percentages for binary and categorical variables. All comparative outcomes will be presented as summary statistics and reported together with 95% CIs.

The PROMIS Upper Extremity Score for Children at three months is the primary outcome of the study and will be compared between treatment groups as the dependent variable in a multivariable linear regression model, adjusting for the stratification factors. An unadjusted *t*-test will also be undertaken. Additional analyses utilizing all the timepoints (from six weeks to one year after randomization) using multilevel modelling will also be undertaken for completeness. Sub-group analysis by fracture type (metaphyseal and Salter-Harris II fractures) will be undertaken using the same methodology by incorporating a treatment by fracture type interaction. Multilevel, mixed-effects, repeated-measures linear regression models will be used to analyze continuous secondary outcomes, if appropriate; otherwise, appropriate non-parametric alternatives will be used. Complications will be reported by type for each treatment group, and, if appropriate, compared between the groups using logistic regression models.

### Health economic evaluation

An economic evaluation of surgery compared with cast immobilization will be conducted from the UK NHS and PSS using the CRAFFT trial data.^31^ A Health Economics Analysis Plan, providing full details of the prospective economic analysis is appended to this document. Health-related quality of life will be estimated using the EuroQol EQ-5D-Y.^18,24^ EQ-5D-Y responses will be valued using the most appropriate valuation set available for the trial population at the time of analysis. If necessary, the adult EQ-5D-3L will be applied, in which case we will undertake sensitivity analysis to make sure that trial findings are not sensitive to the valuation set chosen.^32^ Using the trapezoidal rule, the area under the curve of health status scores will be calculated, providing patient-level quality-adjusted life year (QALY) estimates. Participants’ health service contacts, made in connection with the child’s injury, will be recorded at six weeks, and three, six, and 12 months. Index interventions and subsequent healthcare resource use will be costed using the most recently available published national reference costs, reflated to a common year.^33,34^ Parents’/carers’ out-of-pocket expenses and time lost from work (paid/unpaid) because of their child’s condition, and time off from school, will also be recorded. Resource use questionnaires will be completed by each child’s parent/carer as a proxy response. Mechanisms of missingness of data will be explored, and multiple imputation methods will be applied to impute missing data. Imputation sets will be used in bivariate analysis of costs and QALYs to generate within-trial (12-month) incremental cost per QALY estimates and CIs.^35-38^ Findings will be analyzed and visualized in the cost-effectiveness plane, as cost-effectiveness acceptability curves, net monetary benefit, and value of information analysis. If incremental costs and benefits are non-convergent within the trial follow-up, then extrapolated modelling will be considered, drawing upon epidemiological sources. Sensitivity analyses will be conducted to consider the broader issue of the generalizability of the study results and consider the impact of a broader societal perspective.

### Qualitative study of recruitment and experience of treatment interventions

A qualitative study will be undertaken to identify barriers and facilitators to recruitment. The aim is to increase understanding of the impact of injury and inform practical strategies to improve the process of recruitment in the main trial, for example developments in the presentation of study information. To achieve this, the study will explore: 1) parents’ and children’s experience of injury, treatment, and its impact on their daily life; 2) parents’ and children’s experience of being asked to participate in a randomized controlled surgical trial; and 3) staff experience of being involved in a paediatric surgical trial. To achieve this, qualitative interviews will be incorporated throughout the study. A full description of the qualitative work is available in the complete protocol online on the NIHR website.

### Trial oversight

The trial will be conducted in accordance with the Medical Research Council’s Good Clinical Practice (GCP) principles and guidelines, the Declaration of Helsinki,^39^ OCTRU standard operating procedures (SOPs), relevant UK legislation, and the current approved version of the study protocol. GCP-trained personnel will conduct the trial.

The day-to-day management of the trial will be the responsibility of the trial manager, supported by the OCTRU administrative staff. This will be overseen by the trial management group, who will meet monthly to assess progress. It will also be the responsibility of the Trial Manager to undertake training of the research staff at each of the trial centres. The trial statistician (DK), health economist (JM), and the information specialist (DA) will be closely involved in setting up data-capture systems, design of databases, and clinical reporting forms.

A TSC and a DSMC will be set up. The DSMC will adopt a DAMOCLES charter that defines its terms of reference and operation in relation to oversight of the trial.^40^ They will not be asked to perform any formal interim analyses of effectiveness. They will, however, see copies of data accrued to date, or summaries of that data by treatment group, and they will assess the screening algorithm against the eligibility criteria. They will also consider emerging evidence from other related trials or research, and review related SAEs that have been reported.

### Quality control

This study will be coordinated by the UK Clinical Research Collaboration-registered OCTRU at the University of Oxford. We will institute a rigorous programme of quality control. The trial management group will be responsible for ensuring adherence to the trial protocols at the trial sites. Quality assurance checks will be undertaken by the central trial team to ensure integrity of randomization, study entry procedures, and data collection. The Clinical Trials Unit (CTU) has a quality assurance manager who will monitor this trial by conducting inspections (at least once in the lifetime of the study and more if deemed necessary) of the trial master File. Furthermore, the processes of consent taking, randomization, registration, provision of information, and provision of treatment will be monitored by the central trial team. Written reports will be produced for the TSC, informing them if any corrective action is required.

Additionally, the study may be monitored or audited by sponsor or host sites in accordance with the current approved protocol, GCP, relevant regulations, and SOPs.

### Patient and public involvement

Patients and children were involved from the inception of the trial, including in the development of the funding application. The study was discussed in detail with members of the NIHR Young Person’s Advisory Group,^41^ who chose the logo for the study from an online design competition. An ongoing commitment has been made to continue to work with this group in the production of patient-facing materials and the study dissemination plan.

To ensure ongoing patient and public involvement, a patient/carer representative will be actively involved in general trial management. In addition, a further independent patient/carer representative will become a member of the steering committee.

### Ethics and dissemination

The National Research Ethics Committee approved this study on 16th April 2020 with reference number 20/WM/0054. The full study report for the NIHR Health Technology Assessment will be prepared by the trial management team upon completion of the trial. A manuscript for a high-impact, peer-reviewed journal will be prepared simultaneously, which will allow for the results to be disseminated across the orthopaedic and emergency medicine communities, the wider medical community, the National Institute for Health and Care Excellence, and policy makers. Authorship will be determined in accordance with the International Committee of Medical Journal Editors guidelines, and other contributors will be acknowledged. The results of this trial will substantially inform clinical practice on the clinical and cost-effectiveness of the treatment of this injury. The results of this project will be disseminated to patients via patient-specific newsletters and through local mechanisms at all participating centres.

**Take home message**

- The Children’s Radius Acute Fracture Fixation Trial (CRAFFT) is a multicentre randomized controlled study evaluating surgical versus non-surgical management of displaced distal radius fractures in children.

- The findings may have far-reaching implications for the treatment of distal radius fractures in children.

## Data Availability

The data that support the findings for this study are available to other researchers from the corresponding author upon reasonable request.
